# Performance of MRI-based deep learning models in differentiation of triple negative breast cancer from other breast cancer subtypes: A systematic review and meta-analysis

**DOI:** 10.1016/j.ejro.2026.100808

**Published:** 2026-08-24

**Authors:** Saeed Mohammadzadeh, Iman Kiani, Seyed Amir Mohammad Seyed Rahmani, Moein Mirzai, Mohammadreza Elhaie, Mahsa Esmaeili Dahaj, Mohammad Rahimi, Morteza Mosadegh, Sajjad Mohammadzadeh, Masoumeh Gity

**Affiliations:** a Advanced Diagnostic and Interventional Radiology Research Center (ADIR), Tehran University of Medical Sciences, Imam Khomeini Hospital, Tehran, Iran; b School of Medicine, Tehran University of Medical Sciences, Tehran, Iran; c Iranian Center of Neurological Research, Neuroscience Institute, Imam Khomeini Hospital, Tehran University of Medical Sciences, Tehran, Iran; d Department of Medical Physics, School of Medicine Isfahan University of Medical Sciences, Iran; e School of Medicine, Golestan University of Medical Sciences, Gorgan, Iran

**Keywords:** Triple negative breast cancer, Deep learning, Artificial intelligence, Magnetic resonance imaging, Diagnostic test accuracy, Meta-analysis

## Abstract

**Background and aim:**

Triple negative breast cancer (TNBC) is an aggressive subtype of breast cancer with limited targeted therapies. Deep learning (DL) applied to magnetic resonance imaging (MRI) offers a promising noninvasive alternative to biopsy. This systematic review and meta‑analysis aimed to synthesize current evidence on the diagnostic performance of MRI‑based DL models for identifying TNBC.

**Material and methods:**

Comprehensive searches of PubMed, Scopus, and Web of Science were conducted up to December 5, 2025. Eligible studies evaluated histologically confirmed breast cancer using MRI and DL‑based models for distinguishing TNBC from non‑TNBC. Study quality was assessed using the METRICS. Pooled diagnostic estimates were computed using a bivariate random-effects model in Stata version 18. Sensitivity analyses and publication bias tests were performed. Certainty of evidence was evaluated by GRADE.

**Results:**

Nine studies comprising 2985 patients met inclusion criteria. Pooled estimates in the validation cohorts demonstrated an AUC of 0.85 (95% CI: 0.81–0.88), sensitivity of 0.83 (95% CI: 0.76–0.89), specificity of 0.87 (95% CI: 0.82–0.91), positive likelihood ratio of 6.47 (95% CI: 3.98–10.54), negative likelihood ratio of 0.24 (95% CI: 0.18–0.33), and diagnostic odds ratio of 26.53 (95% CI: 13.86–50.81). Heterogeneity was moderate (I²=30.2%), and no publication bias was detected. Sensitivity analysis revealed no influential individual study. Evidence certainty was rated as low.

**Conclusion:**

DL models applied to breast MRI showed potential for noninvasive TNBC identification with high diagnostic accuracy. However, limited external validation and variability in methodological quality highlight the need for standardized, multicenter studies before clinical implementation can be considered.

## 1 Introduction

Breast cancer is the most commonly diagnosed cancer among women worldwide and remains a major cause of cancer related mortality [1]. Although screening and treatment have improved over time, breast cancer is still a highly heterogeneous disease, with subtypes that show diverse behaviors and varying clinical outcomes [2], [3].

Triple negative breast cancer (TNBC) is one of the most aggressive breast cancer subtypes. It is defined by the absence of estrogen receptor (ER), progesterone receptor (PR), and human epidermal growth factor receptor 2 (HER2) expression, and accounts for about 10–20% of all breast cancer cases. Correctly identifying TNBC is especially important because its treatment options and prognosis are very different from those of other breast cancer subtypes. Unlike hormone receptor positive or HER2 positive tumors, TNBC does not benefit from targeted therapies and is mainly treated with chemotherapy. As a result, early and accurate diagnosis plays a key role in treatment planning and outcome prediction. Failure to correctly distinguish TNBC from other breast cancer subtypes may lead to inappropriate treatment choices and worse outcomes [4].

At present, breast cancer diagnosis and molecular subtyping rely on tissue biopsy followed by histopathological and immunohistochemical analysis [4]. While biopsy is the reference standard, it is invasive and may not fully reflect tumor heterogeneity, as only a small part of the tumor is sampled [5]. In addition, repeated biopsies are not always feasible for monitoring disease progression or treatment response [6]. These limitations have increased interest in noninvasive diagnostic approaches that can provide information about tumor biology without the need for tissue sampling [7].

Magnetic resonance imaging (MRI) is widely used in breast cancer evaluation because of its high soft tissue contrast and functional imaging capabilities [8]. Multiparametric breast MRI, including dynamic contrast enhanced (DCE) imaging and diffusion weighted imaging (DWI), allows noninvasive assessment of tumor vascularity and cellularity. These imaging features are closely related to tumor aggressiveness and may help distinguish TNBC from less aggressive breast cancer subtypes [9]. Compared with mammography and ultrasound, MRI shows higher sensitivity, especially in women with dense breast tissue, and provides quantitative imaging biomarkers that reflect underlying tumor biology [10].

In recent years, artificial intelligence (AI), particularly deep learning (DL), has gained increasing attention in medical imaging [11]. DL models can automatically learn complex patterns from imaging data without the need for manually designed features. Convolutional neural networks (CNNs), which are commonly used in image analysis, have shown strong performance in tasks such as lesion detection, segmentation, and classification [12]. Applied to breast MRI, DL based methods offer a promising noninvasive approach for tumor characterization and subtype differentiation. Several studies have shown that MRI based DL models can accurately distinguish TNBC from other breast cancer subtypes and from benign breast lesions [13], [14]. By analyzing multiparametric MRI data, DL models can capture subtle imaging patterns related to tumor heterogeneity and aggressiveness that may not be easily recognized by radiologists [15]. The use of DL reduces dependence on invasive procedures, supports personalized treatment planning, and enables a more comprehensive assessment of tumor biology [13], [16].

Despite these encouraging findings, existing studies vary widely in MRI protocols, DL model designs, patient populations, and validation methods. This heterogeneity makes it difficult to directly compare results and draw firm conclusions. Moreover, no standardized approach currently exists for applying DL models in clinical practice for TNBC diagnosis. Therefore, the aim of this study is to conduct a systematic review and meta-analysis of MRI based DL methods for the diagnosis of TNBC. By pooling diagnostic performance results from published studies, this work seeks to clarify the current evidence, evaluate the clinical potential of DL assisted breast MRI as a noninvasive diagnostic tool, and identify key areas for future research.

## 2 Method

This systematic review and meta-analysis was conducted in accordance with the comprehensive principles outlined in the Cochrane Handbook for Systematic Reviews of Diagnostic Test Accuracy [17] and reported following the Preferred Reporting Items for Systematic Reviews and Meta-Analyses (PRISMA) 2020 guidelines [18]. In developing and documenting the literature search strategy, the review followed the PRISMA-Search (PRISMA-S) extension [19]. The review protocol was prospectively registered in the open science platform (OSF; https://osf.io/tka86).

### 2.1 Search process

Two reviewers independently designed preliminary search strategies, employing a combination of database-specific controlled vocabulary terms (e.g., MeSH) and relevant free-text keywords tailored to each database. The initial strategies were then critically compared, refined, and consolidated into a unified search strategy for each database. Any inconsistencies or disagreements between reviewers were resolved collaboratively, with the guidance of an experienced medical librarian to ensure accuracy and comprehensiveness (Supplementary Material). The search strategy incorporated the following main keywords: “deep learning,” “MRI,” and “TNBC,” using parentheses and Boolean operators (OR and AND) as appropriate. A comprehensive literature search was performed in PubMed, Scopus, and Web of Science to identify eligible studies published up to December 5, 2025. No language or publication date restrictions were applied. To ensure comprehensive identification of all relevant studies, the reference lists of included studies were examined to capture any potentially eligible articles not retrieved through database searches (backward citation searching). In parallel, Google Scholar was utilized to identify studies citing the included articles (forward citation searching), thereby broadening the scope of retrieval.

### 2.2 Study selection and eligibility criteria

All records retrieved from the database searches were imported into Rayyan software for screening and duplicate removal. After deduplication, two independent reviewers screened the titles and abstracts of all identified studies to assess their potential eligibility. Articles that were considered relevant or potentially relevant by either reviewer were advanced to full text review.

Studies were eligible for inclusion if they enrolled breast cancer patients with histologically confirmed breast cancer subtypes, employed any form of MRI, and implemented DL for feature extraction and model development to differentiate TNBC from other breast cancer subtypes. We excluded studies if they: (I) were review articles, editorials, letters, case reports, animal studies, or conference abstracts; (II) investigated the prognostic application of DL models for predicting TNBC outcomes; (III) did not provide sufficient information to construct a 2 × 2 contingency table for calculating diagnostic performance metrics even after attempts to contact the corresponding author; or (IV) represented grey literature published without a peer-review process.

Full text articles were then independently assessed by the same reviewers according to the predefined inclusion and exclusion criteria. Any disagreements or conflicts that arose during the screening process were resolved through discussion and consensus between the reviewers. To minimize the risk of data duplication arising from overlapping patient populations, all included studies were meticulously cross-checked with respect to author lists, institutional affiliations, and study recruitment periods. When multiple publications originated from the same research group or dataset, preference was given to the publication presenting the largest sample size and the most complete or detailed data to ensure inclusion of the most comprehensive evidence.

### 2.3 Data extraction

Data extraction was independently conducted by two reviewers using a predefined and standardized data extraction sheet, which was first designed based on three included studies, and further refined to the optimum version. Extracted variables encompassed study characteristics (first author, year of publication, country, study design), patient and lesion characteristics (number of patients and breast lesions, number of TNBC and non-TNBC cases, mean or median age, and reported tumor size), as well as MRI related acquisition parameters. MRI specific data included imaging modality, MRI sequences, scanner manufacturer, magnetic field strength.

Comprehensive information on the DL pipeline was also collected, including segmentation method and dimensionality of analysis (2D or 3D), model architecture and type, use of transfer learning versus training from scratch, combination of DL with conventional machine learning based approaches, incorporation of clinical variables, and model validation procedures, encompassing both internal and external validation.

For each eligible model, diagnostic performance metrics were extracted, primarily the area under the receiver operating characteristic curve (AUC). Whenever available, raw diagnostic data, including true positives (TP), false positives (FP), false negatives (FN), and true negatives (TN) were extracted. In studies that did not directly report a 2 × 2 contingency table, the corresponding values were reconstructed using the sensitivity and specificity. For the purpose of this review, TNBC detection was considered the positive outcome; therefore, TP and FN were defined as TNBC cases correctly or incorrectly identified by the DL model, respectively, while FP and TN represented non-TNBC cases incorrectly or correctly classified as TNBC. All data entries were crossverified. Any discrepancies in data extraction were resolved through discussion, and if unresolved, adjudicated by a third reviewer.

### 2.4 Quality assessment

The methodological quality and risk of bias for each included study were independently evaluated by two reviewers using the METhodological RadiomICs Score (METRICS) framework. Any discrepancies between reviewers were initially discussed to reach a consensus and, if disagreement persisted, adjudicated by a third reviewer. The METRICS tool is endorsed by the European Society of Medical Imaging Informatics (EuSoMII) and offers a transparent and standardized approach for the critical appraisal of radiomics and machine learning research. It assesses 30 predefined criteria encompassing key aspects of study design, data handling, and analytic rigor, yielding a cumulative quality score ranging from 0 to 100 [20]. Within this review, the evaluated METRICS domains included imaging protocol and data acquisition procedures, feature extraction and processing methods, validity of statistical analyses and model evaluation strategies, clinical applicability, reproducibility and reliability reporting, and overall methodological design quality. We employed the METRICS checklist rather than the Quality Assessment of Diagnostic Accuracy Studies−2 (QUADAS−2), as it was specifically developed for radiomics and AI-based imaging research. METRICS additionally evaluates domains that are not addressed by QUADAS−2, which was designed for general diagnostic accuracy studies. Importantly, the core QUADAS−2 domains are conceptually embedded within the relevant METRICS items.

### 2.5 Data synthesis

Statistical analyses were conducted following established methodological recommendations for diagnostic test accuracy meta-analyses. Pooled estimates of sensitivity and specificity were obtained using a bivariate random effects model, which appropriately accounts for the inherent correlation between these two parameters and the expected variability across studies. In parallel, pooled positive likelihood ratio (PLR), negative likelihood ratio (NLR), and diagnostic odds ratio (DOR) were calculated, each reported with 95% confidence intervals (CIs). A hierarchical summary receiver operating characteristic (ROC) curve was constructed to visualize the overall diagnostic performance across studies. Between study heterogeneity was evaluated using the I² statistic with values < 50% indicating low heterogeneity. Considering the presence of inter study variability, a random effects approach was applied consistently throughout the analyses. For each eligible study, one representative DL model was selected for meta-analysis according to a predefined selection hierarchy. When available, performance metrics derived from an external validation dataset were prioritized. In the absence of external validation, results from an internal validation subset (either test or hold-out set) were used, while performance obtained from the training set was excluded to avoid overestimation of diagnostic accuracy. In studies that developed multiple models using the same validation dataset, the model explicitly recommended by the authors or the one demonstrating the highest diagnostic performance was chosen. Meta-regression to investigate potential sources of heterogeneity was initially planned; however, as only nine studies were available for pooling, which falls below the minimum of ten studies per covariate recommended by the Cochrane Handbook for reliable meta-regression, this analysis was not performed in order to avoid model overfitting and unreliable inference. Publication bias was assessed using Deeks’ funnel plot asymmetry test. Leave-one-out sensitivity analyses were conducted by sequential omission of individual studies to examine the robustness of the pooled estimates and to identify any study with substantial influence on the overall results. All analyses were performed using Stata software version 18.

## 3 Results

### 3.1 Screening and selection of articles

**Fig. 1:**
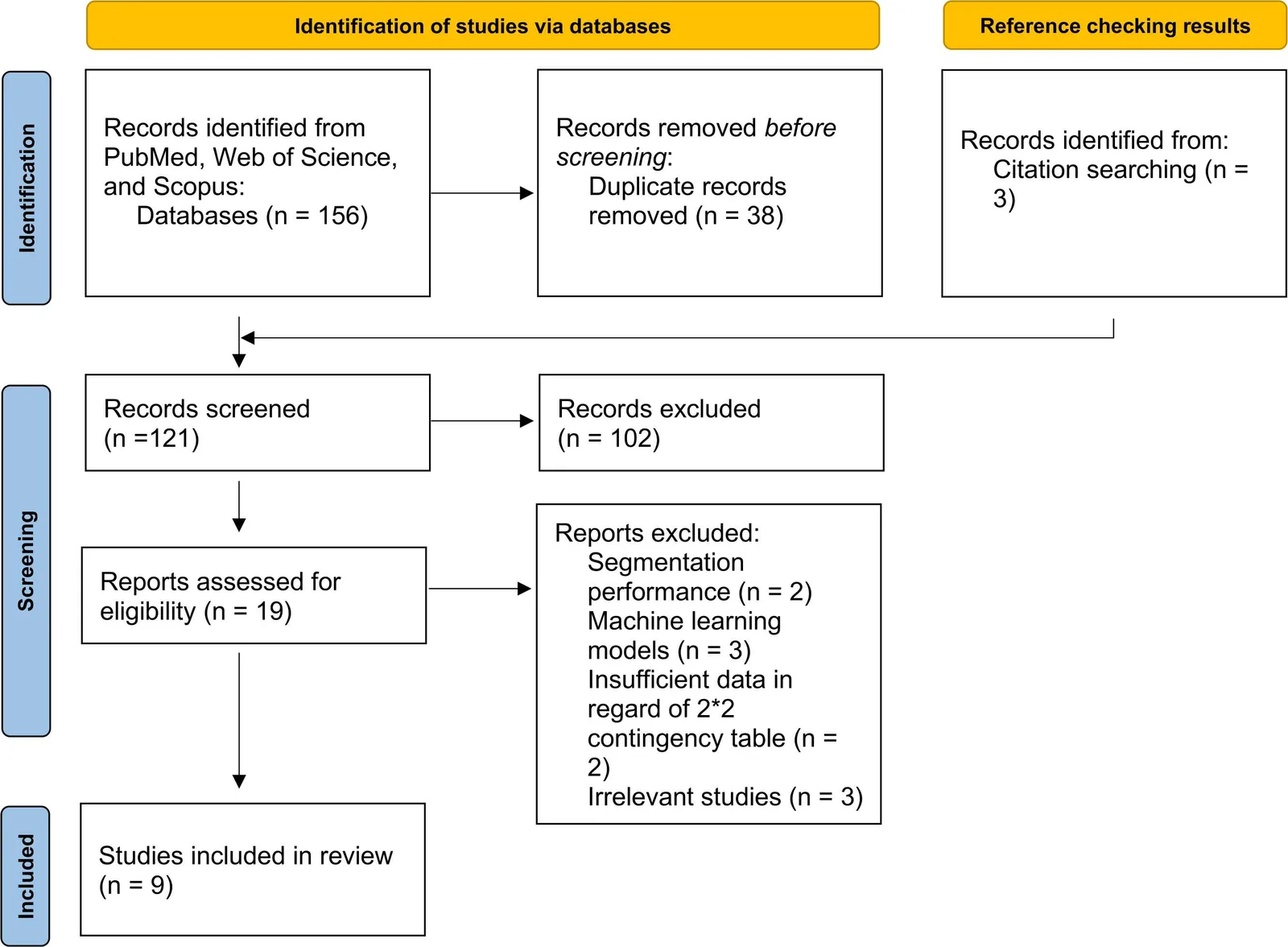
PRISMA flow diagram of study identification, screening, eligibility assessment, and inclusion. PRISMA, Preferred Reporting Items for Systematic Reviews and Meta-Analyses; n, number.

A structured literature search conducted using a predefined strategy initially yielded 156 records. After removing duplicates, 121 unique articles remained for title and abstract screening, during which 102 were excluded as irrelevant. The remaining 19 articles were then assessed for eligibility in the full-text review process. Following this assessment, ten articles were excluded for not meeting the study objectives. Specifically, two articles were excluded due to evaluating the segmentation performance [21], [22], three were removed because they employed machine-learning rather than DL models [23], [24], [25], two were excluded for insufficient data to provide 2*2 contingency table data [14], [26], and three were deemed irrelevant to the study question [27], [28], [29]. Ultimately, 9 studies satisfied all inclusion criteria and were incorporated into the final analysis [12], [30], [31], [32], [33], [34], [35], [36], [37]. Fig. 1 presents the screening process using a PRISMA flow diagram.

### 3.2 Study and patient characteristics

This review includes nine studies in total, of which six employed retrospective designs [30], [31], [32], [34], [35], [36] and three utilized prospective designs [12], [33], [37]. This comprehensive review includes a total of 2985 patients. Among them, 243 were identified as having TNBC, noting that two studies did not report the number of patients in BC subtypes [12], [37]. Most of the studies were conducted in China (n = 7) [12], [30], [31], [32], [33], [35], [37], followed by a single study conducted in United State [36] and another study jointly conducted in both the United States and Taiwan [34]. The studies utilized both single-center (n = 6) [12], [30], [32], [33], [35], [37] and multi-center (n = 3) [31], [34], [36] datasets, with sample sizes ranging from small cohorts of 58 participants [33] to more than 687 patients [31]. Patients were typically around 50 years of age

Concerning imaging methods, the majority of investigations utilized DCE-MRI alone or as part of multiparametric MRI [12], [30], [31], [32], [34], [35], [36], [37]. One study employed synthetic MRI (SyMRI/MAGiC) at 3 T to generate quantitative T1, T2, and PD maps and synthetic contrasts [33]. Two studies incorporated both internal and external validation [31], [34], while the others used internal validation only. The predominant model architectures utilized were convolutional neural networks (CNNs), ResNet-based networks, and multi-branch or ensemble DL models. The majority of investigations employed manual tumor or lesion segmentation [12], [30], [32], [33], [35], [36], [37], while a few studies utilized automatic or semi-automatic methods, including 3D model-assisted or fuzzy C-means-based segmentation [31], [34]. None of the studies employed combined clinical and imaging models. Table 1 and the Supplementary Material summarize the characteristics of the included studies and the technical details of the employed models.

**Table 1:** Characteristics of the nine studies included in this study. TNBC, triple-negative breast cancer; DCE-MRI, dynamic contrast-enhanced magnetic resonance imaging; NME-DWI, non-mass-enhancement diffusion-weighted imaging; IVIM, intravoxel incoherent motion; IDC, invasive ductal carcinoma; ILC, invasive lobular carcinoma; DCIS, ductal carcinoma in situ; HER2, human epidermal growth factor receptor 2; T, Tesla (magnetic field strength); T1WI/T2WI, T1-/T2-weighted imaging; SyMRI, synthetic MRI; MAGiC/MDME, multi-dynamic multi-echo synthetic MRI sequence; DLR, deep-learning reconstruction; mpMRI, multiparametric MRI; ADC, apparent diffusion coefficient; HR + , hormone receptor–positive; METRICS, METhodological RadiomICs Score; mm, millimeter; cm, centimeter; y, years; NR, not reported.

First Author/year	Country	Patient enrollment and publication date	Study design	Number of patients	Number of TNBC patients	Mean/median age	Cancer types	Size of primary tumor	Imaging modality	METRICS Score
Ba et al. (2024)	China	Jul 2018–Sep 2021 (enrollment); 2024 (publication)	Prospective, single-center	475	74	Mean age (y) by subtype: Luminal A 50 ± 5; Luminal B(HER2–) 52 ± 6; Luminal B(HER2 +) 47 ± 10; HER2-enriched 49 ± 9; TN 54 ± 9	(Luminal A, Luminal B HER2–, Luminal B HER2 +, HER2-enriched, TN) + pathologic types (IDC/ILC/mixed/etc.)	Tumor size (mm) by subtype: 34 ± 21; 34 ± 21; 32 ± 21; 33 ± 24; 34 ± 23	DCE-MRI (1 pre + 5 post frames) + NME-DWI (IVIM + diffusion kurtosis + stretched exponential param maps) at 3.0 T Multiparametric MRI (DCE-MRI + NME-DWI)	62.00%
Sun et al. (2023)	China	2017–2019 (enrollment); 2023 (publication)	Retrospective, single-center	160	28	Grouped by ≤ 41 vs > 41	Non-specific invasive breast cancer; subtypes: Luminal A/B, HER2 + , triple-negative	Grouped by tumor size ≤ 20 mm vs > 20 mm	Breast MRI including DCE-MRI (3D VIBRANT); ROI drawn on T1WI, T2WI, and DCE	62.60%
Tang et al. (2025)	China	2025 (publication)	Retrospective, single-center	344	46	mean (range) by subtype: LumA 54.8 (39–78), LumB 55.0 (30-84), HER2 + 52.4 (30–68), TNBS 52.1 (33–64)	Breast cancer molecular subtypes: Luminal A, Luminal B, HER2 + , Basal-like (TNBC); path types incl. IDC/ILC/DCIS/mucinous/papillary	Lesion diameter (mm) by subtype: LumA 2.1(1.0–3.8), LumB 2.0(1–4.9), HER2 + 2.2(1.2–4.5), Basal-like 2.2(1.3–3.4)	1.5 T breast MRI (Philips Achieva); sequences incl. T1WI/T2WI/DWI + DCE e_THRIVE	64.40%
Wang et al. (2025)	China	Jan 2015–Jan 2021 (enrollment); 2025 (publication)	Retrospective, multicenter	687	48	48.70 ± 8.97	Invasive ductal or lobular carcinoma	Tumor size (cm), mean±SD by dataset: 4.20 ± 1.73 (train), 4.20 ± 1.61 (internal test1), 3.73 ± 1.36 (external test2), 4.32 ± 1.64 (external test3)	Breast MRI; classification based on contrast-enhanced MRI (peak enhancement phase selected)	76.30%
Xie et al. (2024)	US	Jan 2000–Mar 2014 (enrollment); 2024 (publication)	Retrospective secondary analysis, multicenter	395	58	NR	Pathologically confirmed breast cancer; luminal A/B, HER2-enriched, TN	NR	DCE-MRI (T1-weighted 4 post-contrast sequences)	58.70%
Yang et al. (2025)	China	2024 Feb–Aug (enrollment); 2025 (publication)	Prospective, single-center	58	10	Mean 50.38 years	Breast cancer (mass-type lesions); carcinoma in situ (n = 3), invasive carcinoma (n = 54), pathology incomplete (n = 1)	NR	3 T breast MRI; SyMRI (MAGiC/MDME): standard 2 × SyMRI vs accelerated 3 × SyMRI vs 3 × SyMRI with DLR (AIR Recon DL); quantitative T1/T2/PD maps + synthetic contrasts	61.90%
Yin et al. (2022)	China	2022	Retrospective, single-center	136	23	Median 51 (IQR 45–57)	Invasive breast cancer (mostly IDC)	TN group: ≤ 2.0 cm 17/23 (73.9%); 2.1–4.0 cm 6/23 (26.1%)	3.0 T mpMRI (T2W, DWI-derived ADC, DCE on T1W)	65.40%
Zhang et al. (2021)	Taiwan/US	Training: Aug 2013–Dec 2014; Testing−1: Jan 2017–May 2018; Testing−2: Jun–Dec 2018 (enrollment); 2021 (publication)	Retrospective, multicenter	244	30 total	Mean age: Training 48; Testing−1 51; Testing−249	Breast cancer subtypes: HR + /HER2 −, HER2 + , TN; counts—Training: 65/24/10; Testing−1: 54/19/10; Testing−2: 37/15/10	Mean tumor size (histology): Training 2.6 cm; Testing−1 2.0 cm; Testing−2 2.1 cm	DCE-MRI only	66.80%
Zhou et al. (2024)	China	2018–2021 (enrollment); 2024 (publication)	Prospective, single-center	486	NR	Mean±SD by subtype (years): LumA 50 ± 5; LumB 52 ± 7; HER2 + 48 ± 11; TN 54 ± 9	Molecular subtypes: luminal A/B, HER2 + , TN; Pathologic types reported (IDC/ILC/mixed/mucinous/others)	NR	3.0 T overall b-value DW-MRI (13 b-values: 0–2000 s/mm²) + DCE-MRI (1 pre + 5 post);	60.60%

### 3.3 Quality assessment

The quality of the models, evaluated using the METRICS checklist, is presented in the Supplementary Material**.** Overall scores ranged from 58.7% to 76.3%, with a mean of 64.3%. Most studies were rated Good [12], [30], [31], [32], [33], [34], [35], [37], with one study rated Moderate [36], according to the checklist. Only one study explicitly states adherence to radiomics and/or machine learning-specific checklists or guidelines [33]. All studies reported eligibility criteria defining a representative study population, employed a high-quality reference standard with a clear definition, and ensured the clinical translatability of the imaging data used for radiomics analysis. Imaging protocols with acquisition parameters were reported in all studies except one [36]. The interval between imaging and the reference standard was explicitly reported in some studies [30], [31], [36], but not in others [12], [32], [33], [34], [35], [37]. Similarly, several studies used test-set segmentation masks generated by a single reader or an automated tool [31], [32], [33], [37], whereas others did not [12], [30], [34], [35], [36]. Only two studies assessed performance relative to non-radiomic approaches or evaluated added clinical value [31], [35]. Several investigations incorporated advanced technical aspects, such as multiparametric imaging [12], [31], [33], [37]. Internal testing was conducted in all studies. However, external validation was conducted in only two studies [31], [34], without providing sufficient data to construct 2 × 2 data highlighting the need for improved generalizability in future research. In the Open Science domain of the checklist, all studies lacked complete code and model transparency, and data availability was rare, with only three studies providing access [30], [35], [36]. Despite most studies achieving a Good rating on reporting standards, limited transparency remains a major barrier to reproducibility and clinical translation.

### 3.4 Meta-analysis of diagnostic accuracy of models in validation cohorts

**Fig. 2:**
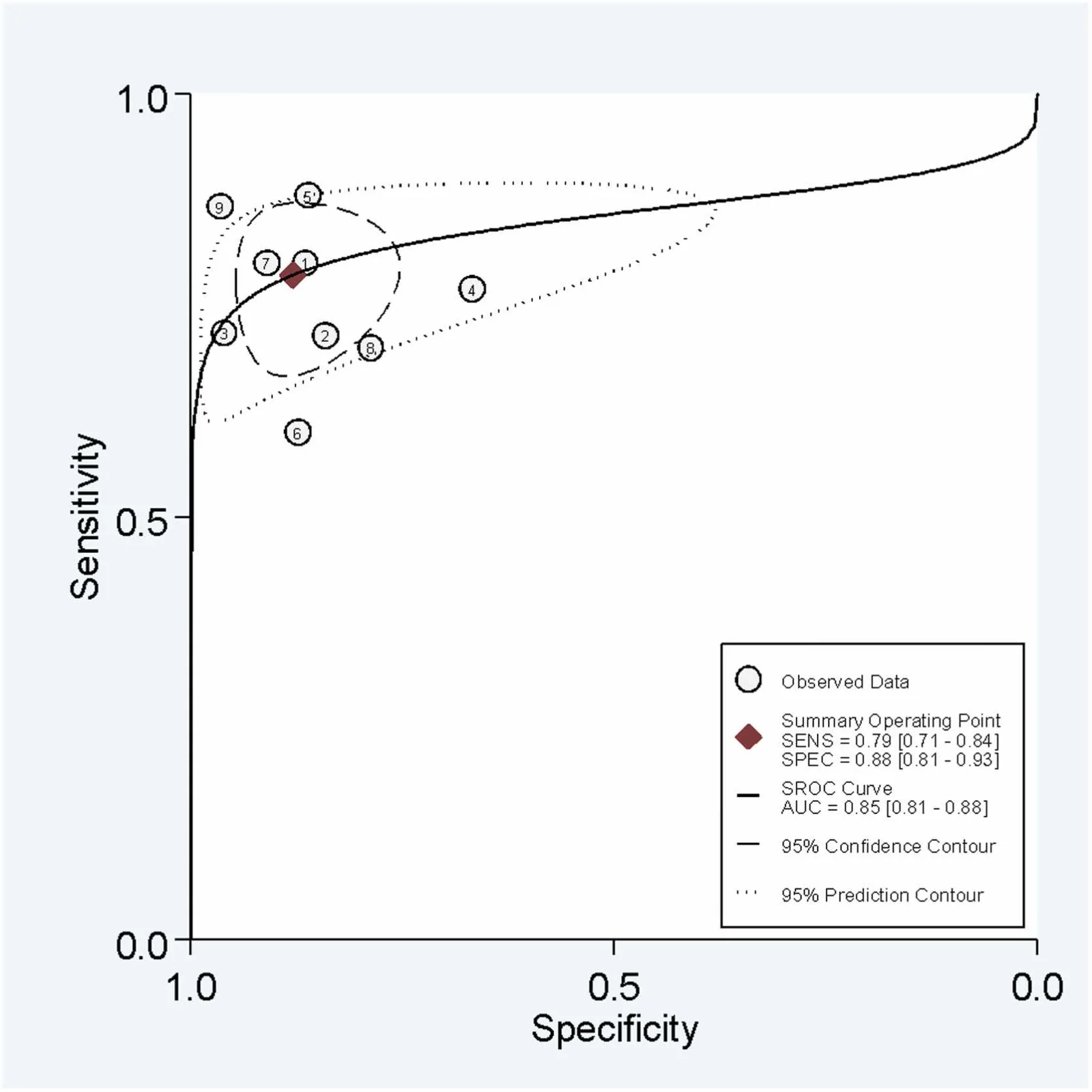
Hierarchical summary receiver operating characteristic (HSROC) curve for the diagnostic performance of deep learning–based MRI models in the validation cohorts of the included studies. Each circle represents the observed sensitivity and specificity of an individual study. The solid point indicates the summary operating point. The solid curve is the summary receiver operating characteristic (SROC) curve. The inner dashed contour is the 95% confidence region around the summary point; the outer dashed contour is the 95% prediction region for a new study. SENS, sensitivity; SPEC, specificity; SROC, summary receiver operating characteristic; AUC, area under the curve; CI, confidence interval.

**Fig. 3:**
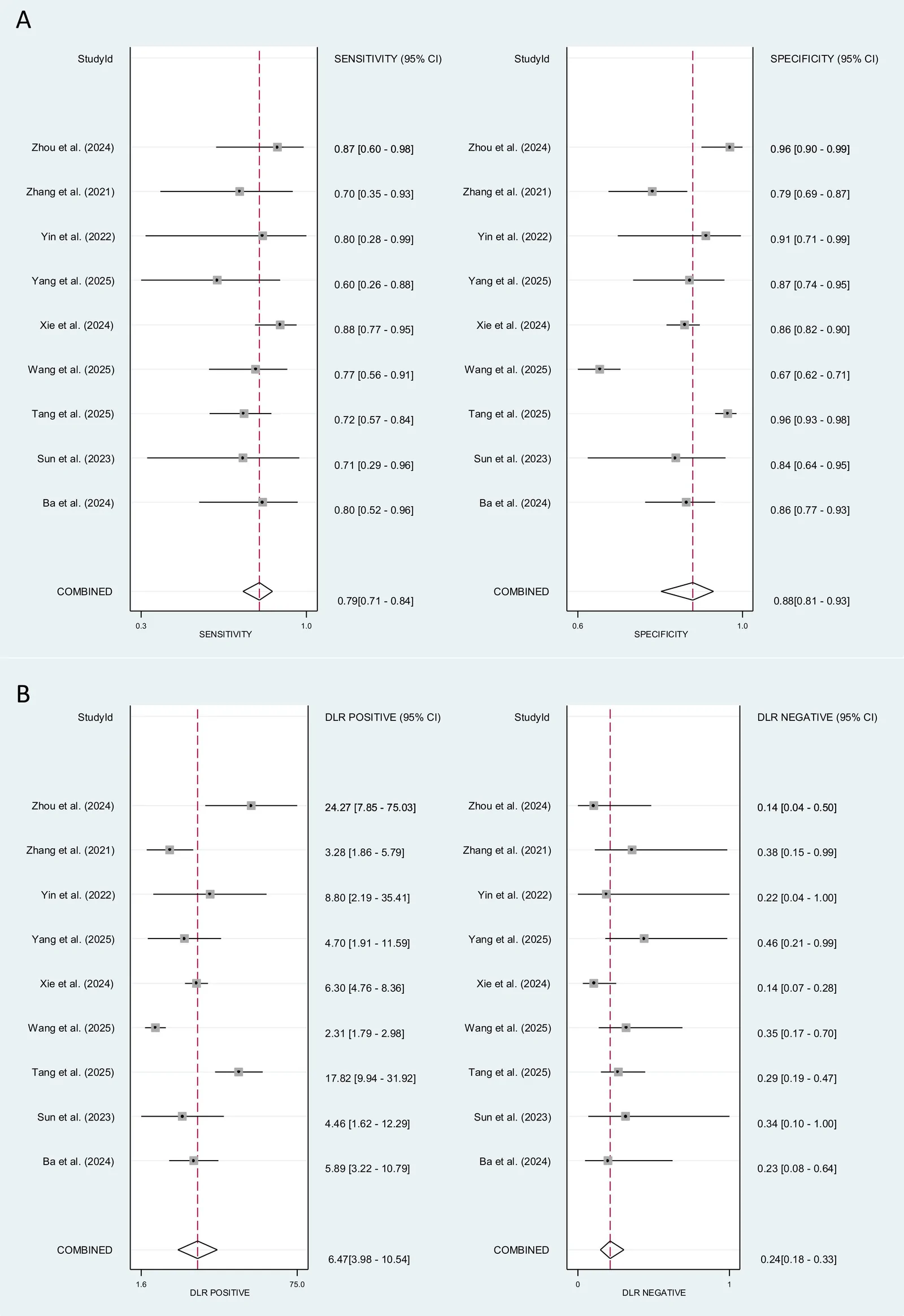

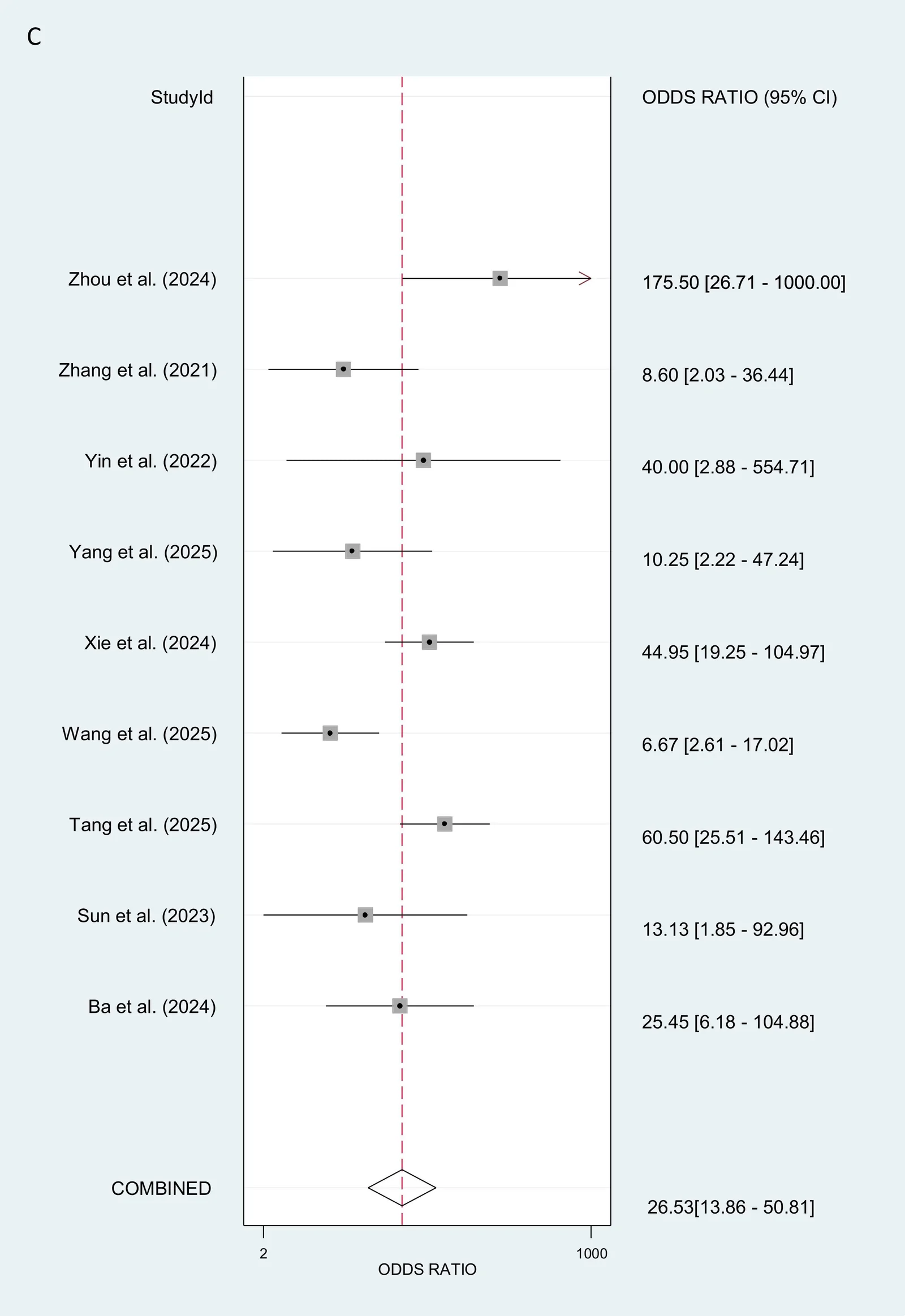
Forest plots of the pooled diagnostic performance of MRI-based deep learning models for differentiating triple-negative breast cancer (TNBC) from non-TNBC using validation cohorts. (A) Forest plots of pooled sensitivity and specificity. (B) Forest plots of pooled positive likelihood ratio (PLR) and negative likelihood ratio (NLR). (C) Forest plot of pooled diagnostic odds ratio (DOR). Each gray square represents the point estimate of the corresponding diagnostic metric for an individual study included with the size of the square proportional to the statistical weight assigned to that study. The horizontal black lines indicate the 95% confidence intervals (95% CIs) for each study estimate. The open diamond at the bottom of each forest plot represents the pooled estimate derived from the bivariate random-effects model; the center of the diamond indicates the pooled effect estimate, while its width corresponds to the 95% CI. The vertical red dashed line denotes the pooled summary estimate and serves as a visual reference for comparison across studies.TNBC, triple-negative breast cancer; CI, confidence interval; PLR, positive likelihood ratio; NLR, negative likelihood ratio; DOR, diagnostic odds ratio.

Despite two studies reporting the presence of external validation cohorts, the complete 2 × 2 data for these external cohorts could not be reconstructed from the published reports. Consequently, all nine cohorts included in our meta-analysis were derived from internal validation sets. Pooled diagnostic metrics from the nine radiomics-only DL-based models demonstrated the following performance: AUC of 0.85 (95% CI: 0.81–0.88), sensitivity of 0.83 (95% CI: 0.76–0.89), specificity of 0.87 (95% CI: 0.82–0.91), PLR of 6.47 (95% CI: 3.98–10.54), NLR of 0.24 (95% CI: 0.18–0.33), and DOR of 26.53 (95% CI: 13.86–50.81), with a generalized I² of 30.2% indicating low heterogeneity. Fig. 2 presents the hierarchical summary ROC curve, while Fig. 3 displays the forest plots for sensitivity, specificity, NLR, PLR, and DOR. Sensitivity analyses showed that no single study influenced the pooled diagnostic estimates (Supplementary Material). The formal assessment of publication bias using Deeks’ funnel plot asymmetry test produced a non-significant result (p = 0.42) (Supplementary Material).

### 3.5 Certainty of evidence

The quality of evidence was appraised using the Grading of Recommendations Assessment Development and Evaluation (GRADE) methodology. Six areas were examined: bias risk in patient enrollment, indirectness/applicability concerns, inconsistency, imprecision, publication bias, and result robustness. Risk of bias was judged high among the selected studies, with some concerns regarding index test reporting and the reporting of the time interval between the index and reference tests. Risk of applicability bias was classified as high, with multiple concerns identified regarding single‑center retrospective designs, lack of external validation, variability in DL pipeline, and heterogeneity in model construction, according to the METRICS evaluation. On inconsistency, statistical heterogeneity remained low. Imprecision was deemed minimal, owing to narrow confidence intervals around pooled AUC values and adequate included study count (n = 9). Publication bias was absent, indicated by insignificant Deeks' test (p > 0.05) and balanced funnel plots in all evaluations. The AUC of 0.85 was interpreted as moderate for the strength of our pooled results. Thus, total evidence certainty was determined low for our study **(**Table 2**)**.

**Table 2:** Summary of the certainty of evidence for the diagnostic accuracy assessed using the GRADE approach across the domains of risk of bias, applicability/indirectness, inconsistency, imprecision, and publication bias. GRADE, Grading of Recommendations Assessment, Development and Evaluation.

Outcome	Number of studies	Number of patients included	Results	Risk of bias	Applicability and indirectness	Inconsistency	Imprecision	Publication bias	Strength of effect size	Certainty
Diagnostic accuracy of deep learning models in validation cohorts	9	2985	Fig. 2 and Fig. 3	High risk	High risk	Consistent	Precise	Low risk	Moderate strength	Low ⊕⊕

## 4 Discussion

This systematic review and meta-analysis provides the first comprehensive synthesis of MRI-based DL models for the noninvasive differentiation of TNBC from other breast cancer subtypes. Pooling data from nine studies encompassing 2985 patients, we found that DL models applied to breast MRI achieve a pooled AUC of 0.85 (95% CI: 0.81–0.88), sensitivity of 0.83, and specificity of 0.87, with a diagnostic odds ratio of 26.53. The reliability of these findings was further supported by low heterogeneity (I²) across studies, no evidence of significant publication bias, and the absence of any influential study in the leave-one-out sensitivity analysis. These findings suggest that DL-based MRI analysis holds potential as a noninvasive adjunct for identifying TNBC, though important methodological limitations temper the strength of this conclusion. It is important to emphasize that the DL models are not yet ready for routine clinical application. Rather than replacing histopathological examination or radiologist interpretation, these tools should be viewed as complementary risk stratification aids that could provide a preliminary risk score to assist in triaging or prioritizing cases particularly in clinical scenarios where radiologist workloads are high, biopsy specimens are insufficient, or a rapid preoperative assessment is needed before definitive pathological evaluation.

The diagnostic performance observed in this meta-analysis compares favorably with that of conventional radiomics approaches. A prior meta-analysis by Sha et al. at 2022 evaluating MRI-based radiomics (without DL) for TNBC diagnosis reported a pooled AUC of 0.87 [16]. While numerically similar, the comparison is instructive: conventional radiomics relies on handcrafted features, such as texture, shape, and intensity-based descriptors, that require manual feature engineering and selection, whereas DL models learn hierarchical representations directly from imaging data [38]. This capacity for automated feature extraction may confer advantages in capturing subtle, nonlinear imaging patterns associated with the aggressive biology of TNBC, including high cellularity, central necrosis, rim enhancement, and rapid washout kinetics on DCE-MRI [39]. That DL models achieve comparable or marginally lower performance to optimized radiomics pipelines likely reflects the relatively small training datasets available in the included studies, a context in which handcrafted features may retain an advantage over data-hungry deep architectures [40].

The pooled sensitivity of 0.83 indicates that approximately one in six TNBC cases would be missed by current DL models. In the clinical context of TNBC, where misclassification may result in the omission of aggressive neoadjuvant chemotherapy [41], this false negative rate warrants careful consideration. Conversely, the specificity of 0.87 suggests that roughly one in eight non-TNBC patients would be incorrectly flagged as TNBC, potentially leading to unnecessary treatment intensification or psychological burden. The positive likelihood ratio of 6.47 and negative likelihood ratio of 0.24 indicate moderate shift in post-test probability, positioning these models as useful triage tools rather than standalone diagnostic tests. In practice, DL-based predictions could serve as a complementary layer of information alongside biopsy results, particularly in clinical scenarios where biopsy is delayed, where tissue is insufficient for complete molecular profiling, or where treatment decisions must be expedited. In this context, we envision DL based MRI classification functioning as a decision support adjunct that complements, rather than replaces, radiologist interpretation and histopathological confirmation.

Several methodological observations across the included studies merit discussion. The predominant use of DCE-MRI reflects its established role in breast cancer assessment and its sensitivity to the neovascularity and perfusion characteristics that distinguish TNBC from other subtypes. TNBC typically exhibits type III washout kinetics, irregular margins, and rim enhancement [42], features that are well captured by dynamic contrast sequences. However, only one study employed synthetic MRI, and the incorporation of DWI, which provides complementary information about tumor cellularity through apparent diffusion coefficient mapping, was limited. Given that TNBC is characterized by high proliferative activity and correspondingly restricted diffusion, the integration of multiparametric MRI sequences into DL pipelines represents an underexplored avenue that could enhance discriminative performance.

To ensure a high degree of methodological homogeneity and robustness in our findings, we applied strict inclusion criteria: our meta-analysis focused exclusively on studies utilizing DL architectures for both feature extraction and model development. By deliberately excluding studies that relied on conventional handcrafted radiomics features or traditional machine learning classifiers (such as SVM, Random Forest, or Logistic Regression) applied to DL-extracted data, we minimized confounding variability. Nevertheless, despite this rigorous selection, the architectural heterogeneity across included models, spanning standard CNNs, ResNet variants, multi-branch networks, and ensemble frameworks, makes it difficult to identify an optimal model design. Beyond model architecture, the included studies also varied in MRI scanner vendor, imaging sequences, magnetic field strength (predominantly 1.5 T and 3 T platforms, with one study employing synthetic MRI reconstruction at 3 T), and in validation strategy. Although the statistical heterogeneity captured by the I² statistic was moderate, this clinical and technical heterogeneity is not fully reflected in I² and should be considered when interpreting the generalizability of our pooled estimates across different clinical settings. Notably, transfer learning was employed in several studies, using pretrained weights from large-scale natural image datasets (e.g., ImageNet) to compensate for the limited size of medical imaging datasets. This strategy is particularly relevant given that the median sample size across included studies was modest [43], with most enrolling fewer than 400 patients and only 243 confirmed TNBC cases across all studies combined. The class imbalance inherent to TNBC classification, where TNBC constitutes only 10–20% of breast cancers [44], poses an additional challenge for model training and may inflate specificity at the expense of sensitivity if not explicitly addressed through oversampling, class weighting, or augmentation strategies [45]. Few of the included studies reported how class imbalance was handled, representing a gap in methodological transparency.

Despite the promising diagnostic performance observed across the included studies, the clinical translation of radiomics-based models for subtype classification remains premature. A critical concern is the absence of external validation, as all cohorts in this meta-analysis were internal validation sets, which primarily reflect overfitting risk rather than true generalizability, particularly in single-center studies where scanner-specific artifacts, institutional imaging protocols, and patient demographics may be inadvertently learned by the model rather than genuine biological signal. The lack of external validation fundamentally limits the generalizability of reported performance metrics and represents the most important barrier to clinical translation [46]. Related to this, none of the included studies combined DL imaging features with clinical variables such as patient age, tumor size, or mammographic density in a multimodal prediction framework. Clinical integration could improve model robustness and facilitate adoption by aligning model inputs with the information already available to treating clinicians.

The quality assessment using the METRICS framework revealed that while most studies achieved "Good" ratings (mean score 64.3%), notable deficiencies persisted. Code and model availability was universally absent, limiting reproducibility. Only one study reported adherence to radiomics- or ML-specific reporting guidelines, and the interval between imaging acquisition and histopathological reference standard was inconsistently documented. These gaps are not unique to the TNBC literature but reflect broader challenges in the radiomics and DL research ecosystem [47], [48], [49]. Encouragingly, no significant publication bias was detected The low heterogeneity (I² = 30.2%) further supports the reliability of the pooled findings.

The geographic concentration of studies in China (seven of nine) raises questions about the generalizability of findings to other populations. Differences in breast density, body habitus, TNBC prevalence, and MRI acquisition protocols across ethnic and geographic groups may influence model performance in ways that cannot be captured by the current evidence [50]. International, multicenter studies enrolling diverse patient populations are needed to establish whether the diagnostic accuracy observed here is externally valid.

This study has limitations. The number of included studies remains small (n = 9), preventing us from conducting meaningful meta-regression and subgroup analyses. Most included studies integrated DCE-MRI with DWI or other sequences within a single pipeline without reporting the independent performance of each sequence, precluding a clean performance assessment for each uni-parametric sequence. The reconstruction of 2 × 2 contingency tables from reported sensitivity and specificity, rather than from raw data, may introduce rounding errors. The exclusion of grey literature publications may have resulted in the omission of relevant evidence, though the absence of detected publication bias is reassuring. Finally, predominance of single-center, retrospective studies without external validation, raising concerns about potential overfitting and the reproducibility of these DL models in broader clinical settings. Prospective multicenter studies with standardized imaging protocols, diverse populations, and robust external validation are urgently needed to translate these deep learning models into clinical practice.

In conclusion, this meta-analysis demonstrates that MRI-based DL models show promising diagnostic accuracy for the noninvasive identification of TNBC, with performance metrics that support their potential role as clinical decision-support tools. However, the current evidence is constrained by small sample sizes, limited external validation, methodological heterogeneity, and geographic concentration. Rigorous, multicenter validation studies with standardized protocols, diverse patient populations, and integration of clinical and multiparametric imaging data are essential to realize the clinical potential of DL-assisted breast MRI in TNBC diagnosis.

## CRediT authorship contribution statement

**Mahsa Esmaeili Dahaj:** Writing – review & editing, Data curation. **Mohammad Rahimi:** Writing – original draft, Investigation. **Moein Mirzai:** Writing – original draft. **Mohammadreza Elhaie Mohammadreza:** Data curation. **Iman Kiani:** Writing – review & editing, Writing – original draft. **Seyed Amir Mohammad Seyed Rahmani:** Data curation. **Saeed Mohammadzadeh:** Writing – review & editing, Writing – original draft, Methodology, Formal analysis, Data curation, Conceptualization. **Mohammadzadeh Sajad:** Writing – review & editing, Visualization, Data curation. **Masoumeh Gity:** Writing – review & editing, Supervision, Project administration, Methodology, Investigation, Conceptualization. **Morteza Mosadegh:** Writing – review & editing, Data curation.

## Declaration of Competing Interest

The authors declare that they have no known competing financial interests or personal relationships that could have appeared to influence the work reported in this paper.
