# Supplementary material for: Performance of MRI-based deep learning models in differentiation of triple negative breast cancer from other breast cancer subtypes: A systematic review and meta-analysis
Source: Eur J Radiol Open. 2026 Aug 24;17:100808. doi: 10.1016/j.ejro.2026.100808 (PMC13542999; doi:10.1016/j.ejro.2026.100808)
Supplement: Supplementary file 1 [file mmc1.docx]

**Supplementary Appendix A:** Search strategy for each database

| PubMed | ("Deep Learning"[Mesh] OR "deep learning"[All Fields] OR "convolutional neural network"[All Fields] OR "deep neural network"[All Fields] OR CNN[All Fields] OR "DL model"[All Fields] OR "neural network"[All Fields] OR LeNet[All Fields] OR AlexNet[All Fields] OR VGGNet[All Fields] OR GoogLeNet[All Fields] OR ResNet[All Fields] OR DenseNet[All Fields])  AND   ("Magnetic Resonance Imaging"[Mesh] OR MRI OR "magnetic resonance imaging" OR "functional MRI" OR "diffusion-weighted imaging" OR "DCE-MRI" OR "multiparametric MRI")  AND  ("Triple Negative Breast Neoplasms"[Mesh] OR "triple negative" OR "triple-negative" OR "TNBC" OR "ER-negative" OR "PR-negative" OR "HER2-negative") |
| --- | --- |
| Web of Science | TS=("deep learning" OR "neural network*" OR "convolutional neural network" OR "deep neural network" OR CNN OR "DL model" OR LeNet OR AlexNet OR VGGNet OR GoogLeNet OR ResNet OR DenseNet)  AND   TS=(MRI OR "magnetic resonance imaging" OR "functional MRI" OR "diffusion-weighted imaging" OR "DCE-MRI" OR "multiparametric MRI")  AND   TS=("triple negative breast cancer" OR "triple negative" OR "triple-negative" OR TNBC OR "ER-negative" OR "PR-negative" OR "HER2-negative") |
| Scopus | ( TITLE-ABS-KEY ( "deep learning" OR "neural network*" OR "convolutional neural network" OR "deep neural network" OR cnn OR "dl model" OR lenet OR alexnet OR vggnet OR googlenet OR resnet OR densenet ) )  AND  ( TITLE-ABS-KEY ( mri OR "magnetic resonance imaging" OR "functional mri" OR "diffusion-weighted imaging" OR "dce-mri" OR "multiparametric mri" ) ) AND  ( TITLE-ABS-KEY ( "triple negative breast cancer" OR "triple negative" OR "triple-negative" OR tnbc OR "er-negative" OR "pr-negative" OR "her2-negative" ) ) |
| PubMed | ("Deep Learning"[Mesh] OR "deep learning"[All Fields] OR "convolutional neural network"[All Fields] OR "deep neural network"[All Fields] OR CNN[All Fields] OR "DL model"[All Fields] OR "neural network"[All Fields] OR LeNet[All Fields] OR AlexNet[All Fields] OR VGGNet[All Fields] OR GoogLeNet[All Fields] OR ResNet[All Fields] OR DenseNet[All Fields])  AND   ("Magnetic Resonance Imaging"[Mesh] OR MRI OR "magnetic resonance imaging" OR "functional MRI" OR "diffusion-weighted imaging" OR "DCE-MRI" OR "multiparametric MRI")  AND  ("Triple Negative Breast Neoplasms"[Mesh] OR "triple negative" OR "triple-negative" OR "TNBC" OR "ER-negative" OR "PR-negative" OR "HER2-negative") |

**Supplementary Appendix B:** Comprehensive METRICS evaluation

**Supplementary Appendix C:** Radiomics pipeline in included studies.

| **Articles** | **Segmentation method** | **Segmentation tool** | **number of features selected** | **cross-validation method** | **transfer learning vs. scratch** | **type of trained models** | **2D or 3D** | **Deep learning alone or Conventional ML + deep learning (fusion model)** | **Deep learning-only or combined model ( deep learning + clinical)** |
| --- | --- | --- | --- | --- | --- | --- | --- | --- | --- |
| Ba et al. (2024) | Manual slice-by-slice segmentation on DW & DCE images after motion correction; ROI propagated; smallest square patch covering ROI extracted; resized to 32×32 | NR (in-house processing; workstation/Python/PyTorch reported) | NA (deep learning feature learning; no manual feature selection) | 20% cases held-out independent test; remaining 80% used for training with 80/20 train-validation split + 5-fold CV | Trained from scratch | DCE-MRI DNN: CNN (6 conv layers) on 32×32×6 patches; global average pooling + FC(5-way) + SoftMax NME-DWI DNN: CNN (6 conv layers) on 32×32×7 patches (7 parametric maps); global average pooling + FC(5-way) + SoftMax MP-MRI ensemble DNN: feature-level fusion (concatenate global features from DCE-MRI and NME-DWI sub-networks → new FC layer); sub-nets first trained, then FC trained with fixed sub-nets, then fine-tuned | 2D | Deep learning alone | Deep learning alone |
| Sun et al. (2023) | Manual ROI outlining by 2 senior breast MRI physicians; cropped bounding box (+10 px margin) and resized to 64×64 | NR | NA (deep learning) | 5-fold cross-validation; train/test split 80/20 (patient-level separation) | Scratch Transfer learning + data augmentation (pretrained on BreakHis) | CAMBNET (cross-attention multi-branch CNN) | 2D | Deep learning alone | Deep learning alone |
| Tang et al. (2025) | Manual slice-by-slice lesion segmentation on contrast-enhanced maps (second DCE frame); 2 radiologists; disagreements resolved by discussion | 3D Slicer v4.10.2 | Radiomics: 110 (no manual selection) ; Deep feature vector: 110; fused: 220 | 5-fold CV; spliting 80% train/20% val) | Transfer learning (fine-tuned ResNet-50) | RAE-Net: ResNet-50 feature extractor + Multi-Head Attention fusion + 3-layer MLP classifier + EDLA uncertainty module | Hybrid: DL uses 2D intensity projections (MaxIP/MinIP/MidIP) ; radiomics includes 3D shape features | Both (radiomics + deep learning + fusion) | Deep learning alone |
| Wang et al. (2025) | Reference standard: manual whole-tumor ROI contours by 2 radiologists (consensus); Automatic model: 3D ResU-Net | Dr. Wise Multimodal Research Platform v1.6.3 (manual ROI creation for reference standard) | NA (deep learning) | Random split (4:1) into train (n=289) and internal test1 (n=61) + 2 external tests (n=73; n=264) Train/internal test split + 2 external test sets (no k-fold CV reported) | Transfer learning (pretrained 2D ResNet used) Scratch (no valid pretrained 3D model; random init) | Classification model: Ensemble ResNet = three 2D ResNet branches (2×ResNet18 + 1×ResNet34) + one 3D ResNet34 branch; averages branch scores Comparator: Radiomics model (handcrafted features + ML | Both 2D & 3D | Deep learning alone Conventional ML | Deep learning alone |
| Xie et al. (2024) | ROI cropping using TCIA-provided rectangular tumor boundaries | TCIA-provided rectangular tumor boundaries | 533 (luminal task); 467 (TN task) | Train/test split 4:1; training split into 10 subsets with 10-fold CV for validation; LASSO uses 10-fold CV (intersection) | Scratch | CORONet: CycleGAN-based cross-sequence joint representation + LASSO feature selection + hypergraph convolution classifier | 2D slices (patient-level voting over slice predictions) | Both (DL + conventional ML feature selection) | Deep learning alone |
| Yang et al. (2025) | Manual ROI delineation (lesion contour at max diameter; gland: 3 circular ROIs) | ITK-SNAP v3.8.0 (ROIs); SyMRI v8.0.4 (map/synthetic image generation) | 3 (mean T1, T2, PD; ROC models use single map) | NR | NA (no model training; vendor DLR pretrained for reconstruction) | ROC classification using T1 map (lesion vs gland) under 2× SyMRI ROC classification using T2 map (lesion vs gland) under 2× SyMRI ROC classification using T1 map (lesion vs gland) under 3× SyMRI ROC classification using T2 map (lesion vs gland) under 3× SyMRI ROC classification using T1 map (lesion vs gland) under 3× SyMRI-DLR ROC classification using T2 map (lesion vs gland) under 3× SyMRI-DLR ROC classification using T2 map (TNBC vs non-TNBC) under 2× SyMRI ROC classification using T2 map (TNBC vs non-TNBC) under 3× SyMRI ROC classification using T2 map (TNBC vs non-TNBC) under 3× SyMRI-DLR | 2D | Deep learning alone | Deep learning alone |
| Yin et al. (2022) | Manual, slice-by-slice whole-tumor ROI | 3D Slicer | NA (end-to-end CNN) | None (single random split) | Transfer learning (ImageNet-pretrained) | ResNet18 binary classifier (TN vs all other subtypes), per-sequence model | 2D (ResNet18; slice/block images resized to 224×224) | Deep learning alone | Deep learning alone |
| Zhang et al. (2021) | 3D tumor ROI on subtraction (2nd DCE frame) using fuzzy C-means; connected-component labeling + hole filling; bounding box from ROI | Fuzzy C-means (FCM) clustering algorithm (in-house process; software NR) | NA (end-to-end DL; no hand-crafted feature selection) | 10-fold cross-validation | Both: scratch (He init) + transfer learning (re-tuning with pre-trained weights from other center) | Conventional CNN Recurrent CNN: CLSTM | 2D | Deep learning alone | Deep learning alone |
| Zhou et al. (2024) | VOI from stacking manually drawn slice-by-slice ROIs; registration across b-values/phases; VOI propagated to other b-values/phases VOI from stacking manually drawn slice-by-slice ROIs; registration across b-values VOI from stacking manually drawn slice-by-slice ROIs; registration across dynamic phases | 3D Slicer (v4.11.20210226) for VOI delineation; MATLAB (R2014a) for subpixel rigid registration 3D Slicer (v4.11.20210226) + MATLAB registration | NA (end-to-end DL; no handcrafted feature selection described) | Independent test set = 20% per subtype; remaining cases split 80/20 train/val; 5-fold CV | Scratch (weights initialized with He Uniform) | DNN | 2D | Deep learning alone | Deep learning alone |

**Supplementary Appendix D:** Sensitivity analysis among validation cohorts of deep learning-based models revealed no influential studies.

**Supplementary Appendix E**: Publication bias among validation cohorts of deep learning-based models revealed no small study effect.
